# New Jersey Leaves No Bite Behind: A Climate Change and Food Waste Curriculum Intervention for Adolescents in the United States

**DOI:** 10.3390/ijerph21040437

**Published:** 2024-04-03

**Authors:** Sara Elnakib, Sabrina Subhit, Jennifer Shukaitis, Amy Rowe, Jeanine Cava, Virginia Quick

**Affiliations:** 1 Department of Family & Community Health Sciences, Rutgers University, New Brunswick, NJ 08901, USA; ss2664@njaes.rutgers.edu (S.S.); shukjen@njaes.rutgers.edu (J.S.); jcava@njaes.rutgers.edu (J.C.); 2 Department of Agriculture & Natural Resources, Rutgers University, New Brunswick, NJ 08901, USA; rowe@njaes.rutgers.edu; 3 Department of Nutritional Sciences, Rutgers University, New Brunswick, NJ 08901, USA; vquick@njaes.rutgers.edu

**Keywords:** climate change education, climate change curriculum, science curriculum, adolescent education, food waste, food waste reduction, greenhouse gas

## Abstract

Food waste is a major contributor to climate change. Schools offer a unique opportunity to educate on this issue while also reducing food waste generation; however, few climate-change education curricula that include a food waste component have been developed and tested with fidelity. Thus, the purpose of this cluster randomized controlled study was to assess the effectiveness of a climate change and food waste education program called NJ Leaves No Bite Behind (NJLNBB) among fifth-grade students. Lessons on food waste and sustainable food behaviors were developed that aligned with NJ Student Learning Standards for Climate Change and Next-Generation Science Standards. Participants (n = 162) completed pre- and post-test surveys that assessed knowledge, attitudes, self-efficacy, and behaviors. Post-test, the experimental group (n = 102) had significantly (*p* < 0.05) higher mean scores in knowledge, social norms, behavioral intentions, and perceived behavioral control compared to the control group (n = 60), with medium effect sizes, as determined by partial eta-squared. There were no significant between-group differences in mean score attitudes, self-efficacy, motivation to comply, or climate-friendly behaviors post-test. Almost three-quarters of participants who received the program agreed or strongly agreed the lessons were fun (75.5%), liked the card games (72.5), and learned a lot (78.4%). These findings are promising in terms of teaching adolescents the impacts of food waste on the climate.

## 1. Introduction

Food waste is a growing social, economic, and environmental issue. In the United States (U.S.), food production accounts for approximately 16 percent of the country’s energy use, nearly 50 percent of land use, and 67 percent of freshwater consumption, yet an astounding 40 percent of the food supply is wasted annually [1,2]. Meanwhile, millions of people in the United States are experiencing food insecurity or lack access to sufficient food to meet their needs. In 2021, 10.2 percent or 13.5 million households experienced food insecurity [3]. Food insecurity is associated with an increased risk of several chronic health conditions, including diabetes, cardiovascular diseases, and mental health disorders [4]. Children who lack access to nutritious foods can face human development implications [4]. Alarmingly, the total dollar loss of food waste is equivalent to USD 473 billion each year in the United States [5]. In addition to the economic losses and consumption of resources associated with food waste, there are significant environmental impacts contributing to climate change. The production, processing, and distribution of food generate carbon dioxide; therefore, wasting food produces harmful greenhouse gases unnecessarily [6]. When food is wasted, it is often sent to landfills where methane, a powerful greenhouse gas, is emitted into the atmosphere. Food waste accounts for 24 percent of all landfill inputs [6].

In a recent assessment of climate-change mitigation approaches, reducing food waste was the highest-impact solution [7]. Schools offer a unique opportunity to reduce food waste generation. A report from the World Wildlife Fund estimates that U.S. schools may generate nearly 530,000 tons of food waste annually, with the equivalent of 1.9 million metric tons of carbon dioxide of greenhouse gases and 20.9 billion gallons of wasted water [8]. According to the U.S. Department of Agriculture, K-12 schools play a special role in food waste reduction while educating the next generation on food conservation and food recovery [9]. Providing adolescents with education on the impact food waste has on the environment may potentially decrease waste generation [10].

Adolescents today are deeply concerned about environmental and social justice issues, especially those related to climate change [11]. While there is agreement among educators, policymakers, NGO and government leaders, and advocates that effective climate change education is required to prepare adolescents to address climate change, effective tools do not currently exist [12,13].

Few interventions in climate change education among adolescents have been published in the literature [14,15,16,17,18,19,20]. The few studies that have been published in the literature have found that climate-change education interventions related to food waste are effective in initiating some degree of behavior change in adolescents. For example, in an educational intervention with 25 adolescents in a primary care school located in Valencia, Spain, there was a 30 percent reduction in food waste post-intervention [14]. Additionally, other climate-change education interventions among adolescent school children report positive changes to students’ food waste knowledge and attitudes [16,19,20] and an increase in the consumption of nutritious foods [14] post-intervention.

According to prior work, the materials and methods used for climate-change education interventions varied. However, these prior studies included some variation of hands-on, interactive, and experiential activities [14,15,16,17,18,19,20]. These activities were either completed individually, in small groups, or in the classroom. In some programs, short educational video lessons were also utilized to capture students’ attention [17,18]. Interestingly, one educational intervention demonstrated the impact of students teaching their peers, which led to a nearly 50 percent reduction in the food waste of salad bar vegetables [19].

Building upon prior work, six lessons on food waste and sustainable food behaviors for NJ schools were developed for adolescents that focused on food systems and food waste. The development of this educational intervention was timely since New Jersey recently became the first state to mandate climate change education in K-12 schools [12]. Given that the existing literature shows the effectiveness of video lessons and in-class, hands-on activities, these lessons included videos, in-class activities, a card game on the food supply chain, and e-learning online games [14,15,16,17,18,19,20]. No study to date has assessed the impact of a curriculum that included all these elements in a multimodal educational program. Lessons were aligned to the NJ’s Student Learning Standards for Climate Change Education [21] as well as the Next-Generation Science Standards [22]. The Theory of Planned Behavior (TPB) was used as a framework to identify key behavioral, normative, and control beliefs affecting climate change behaviors. The TBP encompasses constructs such as knowledge, attitudes, self-efficacy, subjective norms, and perceived behavioral control, which can predict changes in behavioral intentions and behaviors. Thus, the purpose of this study was to assess the effectiveness of a multimodal climate-change education program called New Jersey Leaves No Bite Behind (NJLNBB) in improving climate change knowledge, attitudes, self-efficacy, and behaviors of fifth-grade students (early adolescents ages 10–11 years). It was hypothesized that fifth-grade students who undertook the NJLNBB program would have significantly improved climate change knowledge, attitudes, self-efficacy, and behaviors post-test compared to the control group.

## 2. Materials and Methods

To evaluate the effectiveness of the NJLNBB program in improving climate change knowledge, attitudes, self-efficacy, and behaviors among fifth-grade students (10–11 years of age), a cluster randomized controlled study design was implemented. This study design was approved by the Institutional Review Board at Rutgers University (Pro2021002505). Parental consent was obtained from participants prior to implementing the intervention.

### 2.1. Sample

**Figure 1:**
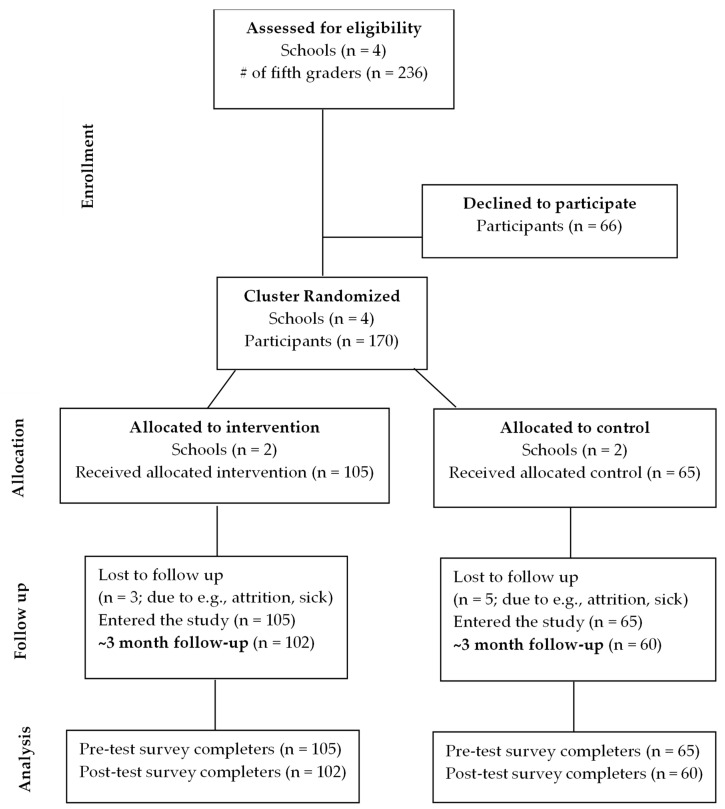
CONSORT for NJLNBB Program.

Four New Jersey schools in two counties volunteered to participate in this study (Figure 1). Schools in each location were carefully matched on school size, student race/ethnic background, and percentage of students receiving free school lunches. One NJ school from each county was randomly assigned to the intervention while the other school was assigned to the control group. The control groups had a delayed intervention, receiving the intervention after the study was completed. Fifth-grade classes were chosen for this study because this is when students in the U.S. begin to learn more in-depth information about the ecosystem as part of their common core education requirements [11]. Additionally, studies indicate that 10–12-year-old children can feel a sense of empowerment and motivation to work on social and environmental challenges [23,24]. As depicted in Figure 1, of the 236 students from 4 schools with fifth graders (ages 10–11 years) that met the study’s eligibility criteria to participate, 170 students received parental consent to participate. Of these, 162 completed both the pre- and post-test surveys. The final sample was 162 participants, with 102 in the experimental group and 60 in the control group. 

### 2.2. Curriculum

The NJLNBB program consisted of six lessons, each about 45 min long. Each lesson consisted of an informative video, PowerPoint slides to disseminate educational information, and a hands-on activity and/or e-learning game where students could apply what they learned. The lessons were taught between January and February of 2023 by a team of nine undergraduate students from Rutgers University. Before the NJLNBB program started, the undergraduate students attended a training session to prepare them for teaching adolescents in classrooms. The project team held weekly Zoom sessions with the students to ensure that they were supported as they taught the NJLNBB program. Table 1 provides the lesson topics covered in the curriculum, including learning objectives and links with the next-generation science standards.

### 2.3. Instruments

To evaluate the effectiveness of the NJLNBB curriculum, all participants completed pre- and post-test surveys using a paper/pencil format in the classroom. An adaptive version of the Theory of Planned Behavior (TPB) was used as a framework, which aided in the design of the survey. The TPB is one of the most popular social-psychological models for predicting behavior [22] and has been widely used in food waste research [25]. All questions on the surveys were created de novo (see Appendix A for the survey items and scales). The online survey was cognitively tested with fifth-grade students (N = 5) and assessed for face validity with content experts (N = 3) on the Theory of Planned Behavior constructs prior to implementation. Experts on survey development were involved in all phases of survey development (i.e., question development, content analysis, cognitive interviews, and pilot testing). Both the pre- and post-test surveys had the following outcome variables, which are the Theory of Planned Behavior constructs, as described below.

Participants’ knowledge of climate change concepts including the food system, food waste, food miles, and composting concepts were assessed by 10 multiple-choice items. A percentage of total knowledge scores from all questions answered correctly was calculated, with higher scores indicating greater knowledge of climate change concepts.

Social norms (four items), attitudes (six items), and self-efficacy (four items) scales were measured on a five-point Likert scale (i.e., 1 = strongly disagree, 2 = disagree, 3 = undecided, 4 = agree, 5 = strongly agree). All items were averaged, with higher mean scores indicating greater influence received from others to engage in climate-friendly behaviors (social norms), positive attitudes toward climate-friendly behaviors (attitudes), and greater confidence toward adopting climate-friendly behaviors (self-efficacy).

A total of six items on a five-point Likert scale (i.e., 1 = very unlikely, 2 = unlikely, 3 = undecided, 4 = likely, 5 = very likely) assessed behavioral intentions. Items were averaged, with higher means scores indicating greater intentions toward adopting climate-friendly behaviors.

Perceived behavioral control (five items) was assessed on a five-point Likert scale (i.e., 1 = very difficult, 2 = difficult, 3 = neither difficult nor easy, 4 = easy, 5 = very easy) All items were averaged, with higher mean scores indicating greater perceived ease of control over adopting climate-friendly behaviors.

Motivation to comply (five items) was assessed on a five-point scale (i.e., 1 = not at all, 2 = slightly, 3 = moderately, 4 = very, 5 = extremely) All items were averaged, with higher mean scores indicating greater motivation towards adopting climate-friendly behaviors.

Climate-friendly behaviors (six items) were assessed on a five-point frequency scale (i.e., 1 = never, 2 = a little, 3 = sometimes, 4 = often, 5 = almost always). All items were averaged, with higher means scores indicating a greater frequency of engaging in climate-friendly behaviors.

Demographic Characteristics. On the pre-test survey, participants self-reported their age, gender, ethnicity, and racial category. Participants had the option to not respond to any of the demographic questions.

Program Satisfaction. On the post-test survey, only the experimental group was asked to respond to eight questions on a five-point Likert scale (i.e., 1 = strongly disagree, 2 = disagree, 3 = undecided, 4 = agree, 5 = strongly agree) that assessed their satisfaction with the NJLNBB program (e.g., “The NJLNBB lessons were fun.”). Additionally, one item asked participants to report how many times they played the NJLNBB online games, and another item inquired whether they showed the online games with their friends or family. The last question asked the experimental group to comment on what they thought about the NJLNBB program.

### 2.4. Data Analysis

Demographic characteristics and study outcome measures by treatment group were analyzed with descriptive statistics. The internal consistency reliability of outcome variables was assessed using Cronbach’s alpha. Analysis of Covariance (ANCOVA) controlling for baseline scores with Bonferroni correction examined significant differences post-test in all study outcome variables (i.e., knowledge, social norms, attitudes, self-efficacy, behavioral intentions, perceived behavioral control, motivation to comply, climate-friendly behaviors) between control and experimental groups. Partial eta-squared indicated small (η^2^ = 0.01), medium (η^2^ = 0.06), and large effect (η^2^ = 0.14) sizes of significant (*p* < 0.05) ANCOVA findings. All analyses were performed in the IBM Statistical Package for Social Sciences (Chicago, IL, USA, version 28).

## 3. Results

The final sample of fifth-grade students had a higher proportion of girls (53.6%) (Table 2). For participants who reported their race (n = 112) and ethnicity (n = 129), most identified as being White (75.9%) and non-Hispanic (68.0%). In general, the control group had a higher proportion of White participants compared to the experimental group (93.8% vs. 68.8%, respectively).

The internal consistency reliability of study outcome variables as determined by Cronbach alpha coefficients ranged from poor to fair (Table 3). The examination of between-group differences in study outcomes post-test, controlling for pre-test scores, revealed significant differences in knowledge, social norms, behavioral intentions, and perceived behavioral control (Table 3). That is, post-test the experimental group had significantly higher mean scores in knowledge, social norms, behavioral intentions, and perceived behavioral control compared to the control group with medium effect sizes as determined by partial eta-squared for all outcomes, except social norms had a small effect size. There were no significant between-group differences in attitudes, self-efficacy, motivation to comply or climate-friendly behaviors mean scores post-test.

Post-test, responses to items on participants’ satisfaction with the NJLNBB program, as shown in Table 4, were rather favorable in the experimental group (n = 102). That is, almost three-quarters of the participants who undertook the NJLNBB program agreed or strongly agreed that the lessons were fun (75.5%), they liked the card games (72.5), and they learned a lot (78.4%). Over half of the participants also agreed or strongly agreed that they wanted to learn more about climate change (65.6%), wanted to teach others about climate change (50.0%), and believed their friends in other schools would learn a lot from the program (71.6%). Additionally, over one-third (39.0%) reported sharing the NJLNBB online games with either their friends or family.

## 4. Discussion

The present study addressed the pressing issue of food waste and its environmental implications, focusing on the effectiveness of a multimodal climate-change education program, New Jersey Leaves No Bite Behind (NJLNBB), in fifth-grade students. While there are some published studies that have evaluated climate change education interventions in children and adolescents [14,15,16,17,18,19,20] there is a gap in theory-based, comprehensive, multimodal educational interventions in the existing literature. As hypothesized, the experimental group that undertook the NJLNBB program had significantly higher mean scores in climate change knowledge, social norms, behavioral intentions, and perceived behavioral control compared to the control group. However, students’ attitudes, self-efficacy, motivation to comply, or climate-friendly behaviors did not change significantly post-test compared to the control group. The post-test survey results regarding program satisfaction provided valuable insights into the students’ experiences with the NJLNBB program. The overwhelmingly positive responses regarding the program’s fun and educational aspects, as well as the expressed interest in learning more about climate change and teaching others, suggest a high level of engagement and enthusiasm among participants.

While climate change education more broadly is important, the findings of this study contribute to the larger context of addressing food waste, aligning with the global push for sustainable practices. Lessons on the economic losses, environmental impacts, and potential contribution to climate change underscore the urgency of effective strategies adolescents can pursue. Similar to a few studies [16,17,18,19,20], this study specifically targeted fifth-grade students, recognizing the importance of early education in shaping environmentally conscious behaviors. While this study did not follow students over a long period of time, the results of the study indicated positive outcomes in several key areas. The experimental group, exposed to the NJLNBB program, exhibited significant improvements in knowledge, social norms, behavioral intentions, and perceived behavioral control compared to the control group. This suggests that the program was successful in enhancing students’ understanding of climate change concepts, influencing their social norms, and fostering intentions and perceived control over climate-friendly behaviors. Additionally, findings indicate the relevance of the age group targeted by NJLNBB.

As mentioned earlier, this study’s design, incorporating a multimodal educational program aligned with New Jersey’s educational standards, demonstrated a thorough and systematic approach. By integrating videos, in-class activities, card games, and e-learning online games, the NJLNBB program aimed to engage students through various learning modalities. This comprehensive design was a novel approach, considering that prior interventions often focused on specific elements such as video lessons [17,18] peer instruction [19], and parental involvement [14,15,17].

Additionally, ensuring assessment measures are relevant to appropriate learning theories is critical to successfully evaluating program outcomes. Many of the prior educational programs did not utilize a theory-based educational framework, and those that did reported non-significant findings in climate change behaviors between the control and intervention groups [18,19]. These findings highlight the need to utilize theoretical frameworks as a critical component of the program design and evaluation. Theories such as the Self-Determination Theory [25] may be intuitively connected to food waste education, but other constructs from the Theory of Planned Behavior [26,27] may be more relevant for adolescents.

Furthermore, this study had a sufficient sample size, like other studies [15,16,17]. However, unlike other studies, demographic characteristics, such as gender and race/ethnicity, were considered in the analysis, ensuring a comprehensive understanding of the study participants. The control group had a higher proportion of White participants compared to the experimental group (93.8% vs. 68.8%, respectively) in this study. Thus, it may be important to consider demographic factors in the interpretation of results and the potential need for tailored interventions.

This study acknowledges several limitations, which may have led to the restricted findings presented. As with all survey-based research, self-reported data may have several biases, including social desirability and response biases. Additionally, self-reported data may not capture unconscious or subconscious factors that influence behavior or decision-making. That is, individuals may not be fully aware of certain motivations or influences on their beliefs and behaviors. Furthermore, this study did not follow participants over a long follow-up period to assess the long-term impacts of the NJLNBB program. Finally, despite this study being a multi-model well-designed educational intervention, it did not include a component for parental involvement as prior studies did [14,15,17].

Future studies would benefit from exploring the sustainability of behavior change over time through a longitudinal study and considering additional factors that may influence outcomes, such as socioeconomic status and parental involvement. Additionally, measuring unintended changes to behavior in future studies, such as increased fruit and vegetable intake, can assess the additional impacts of a comprehensive climate-change education program. Finally, assessing actual climate-friendly behavior changes in addition to knowledge, social norms, behavioral intentions, and perceived behavioral control can create a definitive link between these constructs and the implementation of strategies to impact climate change.

## 5. Conclusions

In conclusion, this study addressed a critical gap in the literature by evaluating the impact of a comprehensive climate-change education program on fifth-grade students. The positive outcomes observed in knowledge, social norms, behavioral intentions, and perceived behavioral control highlight the potential of well-designed educational interventions to contribute to broader efforts in mitigating climate change through informed and environmentally responsible behaviors. The study’s findings have important implications for climate change education, emphasizing the effectiveness of multimodal programs in influencing knowledge, attitudes, and behaviors. Educators, policymakers, and curriculum developers may find these results helpful for informing the design and implementation of climate-change education initiatives, especially those targeting younger age groups.

## Figures and Tables

**Table 1:** NJLNBB curriculum with NJ Next-Generation Science Standards.

Title	Next-Generation Science Standards	Disciplinary Core Ideas	Learning Objectives
Lesson 1: Essential Earth Knowledge: Atmosphere, Greenhouse Gases, and the Climate	5-ESS3-1—Obtain and combine information about ways individual communities use science ideas to protect the Earth’s resources and environment. 5-ESS2-1—Develop a model using an example to describe ways the geosphere, biosphere, hydrosphere, and/or atmosphere interact. 5-LS2-1—Develop a model to describe the movement of matter among plants, animals, decomposers, and the environment.	ESS2.A: Earth Materials and Systems ESS3.C: Human Impacts on Earth Systems	Recall that earth’s atmosphere is a component of an inter-connected climate system. Name three gases in our air, including at least one greenhouse gas. Identify two activities that currently cause the emission of greenhouse gases. Describe two things that can reduce GHG emissions.
Lesson 2: Getting to Know the Food System	5-ESS3-1—Obtain and combine information about ways individual communities use science ideas to protect the Earth’s resources and environment. 5-LS2-1—Develop a model to describe the movement of matter among plants, animals, decomposers, and the environment.	ESS2.A: Earth Materials and Systems ESS3.C: Human Impacts on Earth Systems	Recall the components of the food system. Name three resources that the food system requires.
Lesson 3: Food Waste in the Food System	5-ESS3-1—Obtain and combine information about ways individual communities use science ideas to protect the Earth’s resources and environment. 4 ESS3-2—Generate and compare multiple solutions to reduce the impacts of natural Earth processes and climate change have on humans.	LS2.B: Cycles of Matter and Energy Transfer in Ecosystems ESS2.A: Earth Materials and Systems ESS3.C: Human Impacts on Earth Systems	Identify two sectors or components of the food system where food waste occurs. Describe two things that can help reduce food waste.
Lesson 4: Environmental Impacts of Food Waste and Solutions to Food Waste	5-ESS3-1—Obtain and combine information about ways Individual communities use science ideas to protect the Earth’s resources and environment.	ESS3.C: Human Impacts on Earth Systems	Name all six resources that are wasted when food waste. happens. Describe how food waste contributes to climate change. Identify two ways to reduce food waste.
Lesson 5: Food Miles, Shrinking our Food’s Carbon Footprint	5-ESS3-1—Obtain and combine information about ways individual communities use science ideas to protect the Earth’s resources and environment.	ESS3.C: Human Impacts on Earth Systems	Explain how food miles in connected to global climate change. Identify two foods that are in season in New Jersey that they would try. Describe two ways that they can reduce GHG emissions using knowledge on food miles. Identify where fresh produced was grown by reading labels.
Lesson 6: Composting: Decomposition Helps Us Solve Climate Change!	5-LS2-1—Develop a model to describe the movement of matter among plants, animals, decomposers, and the environment.	LS2.B: Cycles of Matter and Energy Transfer in Ecosystems	Describe the movement of matter among plants, animals, decomposers, and the environment. Explain how matter from our food waste can be returned to the soil to nourish new life. Identify the benefits of composting food waste instead of throwing it in the trash/sending it to a landfill. Distinguish between materials that can be composted.

**Table 2:** Demographic Characteristics by Treatment Group.

	All N = 162	Control n = 60	Experimental n = 102
	N (%)	N (%)	N (%)
School			
Robert L. Horbelt Intermediate	63 (38.9)	-	63 (61.8)
Paterson	39 (24.1)	-	39 (38.2)
Berkely Twp.	39 (24.1)	39 (65.0)	-
Passaic Gifted and Talented	21 (13.0)	21 (35.0)	-
Gender ^a^			
Boy	70 (45.8)	22 (37.9)	48 (50.5)
Girl	82 (53.6)	36 (62.1)	46 (48.4)
Non-Binary	1 (0.7)	0 (0.0)	1 (1.1)
Race ^b^			
White	85 (75.9)	30 (93.8)	55 (68.8)
Asian	11 (9.8)	0 (0.0)	11 (13.8)
Black or African American	10 (8.9)	1 (3.1)	9 (11.3)
Two or more races	6 (5.4)	1 (3.1)	5 (6.3)
Hispanic (% yes) ^c^	49 (38.0)	20 (43.5)	29 (34.9)

^a^ Missing nine responses. ^b^ Missing 50 responses. ^c^ Missing 33 responses.

**Table 3:** Study Outcomes: Baseline Post-Measurement Completers (N = 162).

			Control Group (n = 60)		Experimental Group (n = 102)		ANCOVA between Group Differences Over Time ^a^		
		N = 162	Baseline	Post	Baseline	Post			
Measure (Total Possible Score Range)	# Items	Cronbach’s α	Mean ± SD	Mean ± SD	Mean ± SD	Mean ± SD	*F*	*p*	Partial Eta-Squared
Knowledge (0 to 100) ^b^	10	0.47	50.78 ± 19.06	52.25 ± 17.93	54.76 ± 20.36	66.04 ± 21.64	12.77	<0.001	0.078
Social Norms (0 to 5) ^c^	4	0.43	3.54 ± 0.59	3.62 ± 0.77	3.73 ± 0.57	3.93 ± 0.71	4.07	0.045	0.025
Attitudes (0 to 5) ^d^	6	0.56	3.51 ± 0.58	3.49 ± 0.66	3.78 ± 0.51	3.85 ± 1.04	1.82	0.180	0.011
Self-Efficacy (0 to 5) ^e^	4	0.65	3.54 ± 0.78	3.42 ± 0.90	3.68 ± 0.64	3.59 ± 0.85	0.37	0.543	0.002
Behavioral Intentions (0 to 5) ^f^	6	0.72	3.42 ± 0.71	3.27 ± 0.88	3.56 ± 0.63	3.75 ± 0.64	15.66	<0.001	0.090
Perceived Behavioral Control (0 to 5) ^g^	5	0.59	3.53 ± 0.64	3.33 ± 0.73	3.58 ± 0.59	3.72 ± 0.60	16.46	<0.001	0.095
Motivation (0 to 5) ^h^	5	0.64	3.33 ± 0.63	3.34 ± 0.93	3.55 ± 0.82	3.52 ± 0.81	0.14	0.714	0.001
Engagement with Climate-Friendly Behaviors (0 to 5) ^i^	6	0.77	2.87 ± 0.81	2.81 ± 0.93	2.84 ± 0.81	2.97 ± 0.78	2.20	0.140	0.015

Note: For Partial Eta-Squared, η^2^ = 0.01, 0.06, and 0.14 indicate small, medium, and large effect sizes, respectively. ^a^ Analysis of Covariance controlling for baseline scores with Bonferroni correction. ^b^ 153 participants (n = 56 control; n = 97 experimental) had 8 or more responses for average total knowledge score; higher scores indicate greater knowledge of climate change and food waste concepts. ^c^ 161 participants (n = 60 control; n = 101 experimental) had 3 or more responses for average social norms score; higher scores indicate greater influence received from others to engage in climate-friendly behaviors. ^d^ Higher scores indicate positive attitudes towards climate-friendly behaviors. ^e^ Higher scores indicate greater confidence toward adopting climate-friendly behaviors. ^f^ Higher scores indicate greater intentions towards adopting climate-friendly behaviors. ^g^ 158 participants (n = 57 control; n = 101 experimental) had 5 or more responses for average perceived behavioral control scores; higher scores indicate greater perceived ease of control over adopting climate-friendly behaviors. ^h^ 156 participants (n = 56 control; 100 experimental) had 4 or more responses for average motivation scores; higher scores indicate greater motivation towards adopting climate-friendly behaviors. ^i^ 152 participants (n = 56 control; n = 96 experimental) had 5 or more responses for average scores; higher scores indicate a greater frequency of engaging in climate-friendly behaviors.

**Table 4:** Study Participants’ Satisfaction with the NJLNBB program (n = 102).

Post-Test Questions ^a^	Mean ± SD	% Agree or Strongly Agree
The NJLNBB lessons were fun.	3.95 ± 0.97	75.5
I liked the NJLNBB card games.	3.97 ± 0.97	72.5
I liked the NJLNBB online games.	3.83 ± 0.96	63.8
I learned a lot from participating in this program.	4.01 ± 0.95	78.4
I want to learn more about climate change.	3.75 ± 1.11	65.6
I want to teach others about climate change.	3.45 ± 1.06	50.0
My friends in other schools would like this program.	3.51 ± 1.14	50.0
My friends in other schools would learn a lot from this program.	3.95 ± 1.06	71.6
How many times did you play the NJLNBB online games?	3.01 ± 2.23	-
Did you show the NJLNBB online games to your friends or family? (% yes)	39.0%	-

^a^ Responses to questions were on a five-point Likert scale (1 = strongly disagree, 2 = disagree, 3 = undecided, 4 = agree, 5 = strongly agree).

**Figure A1:**
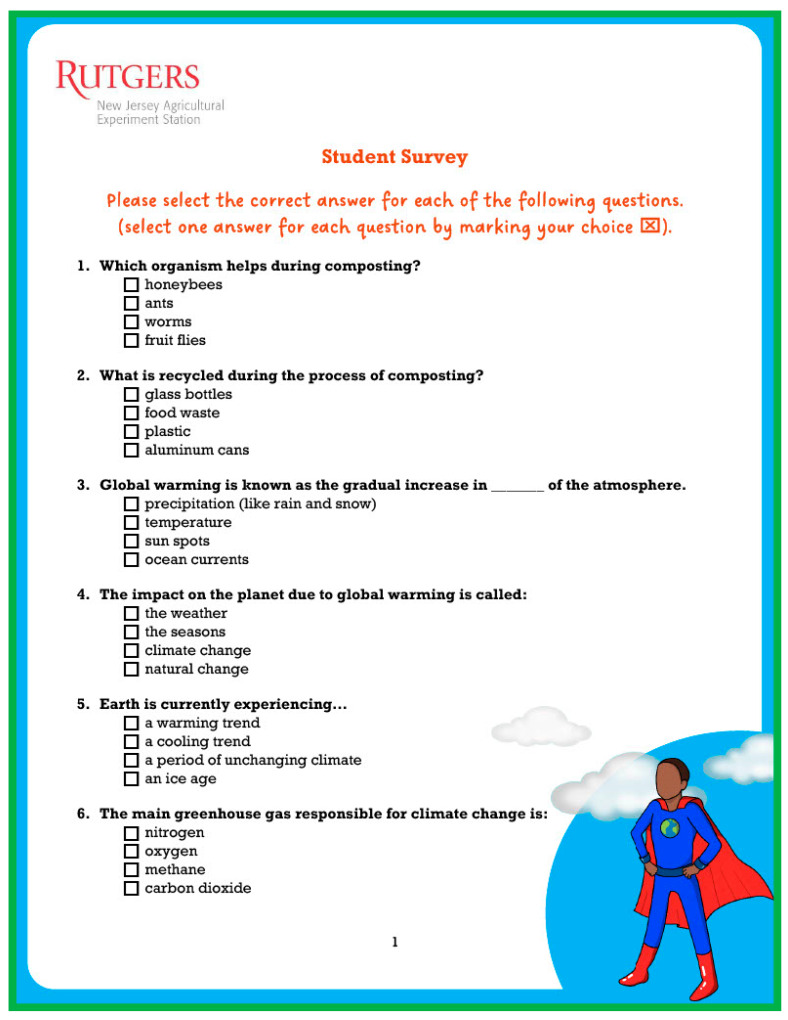

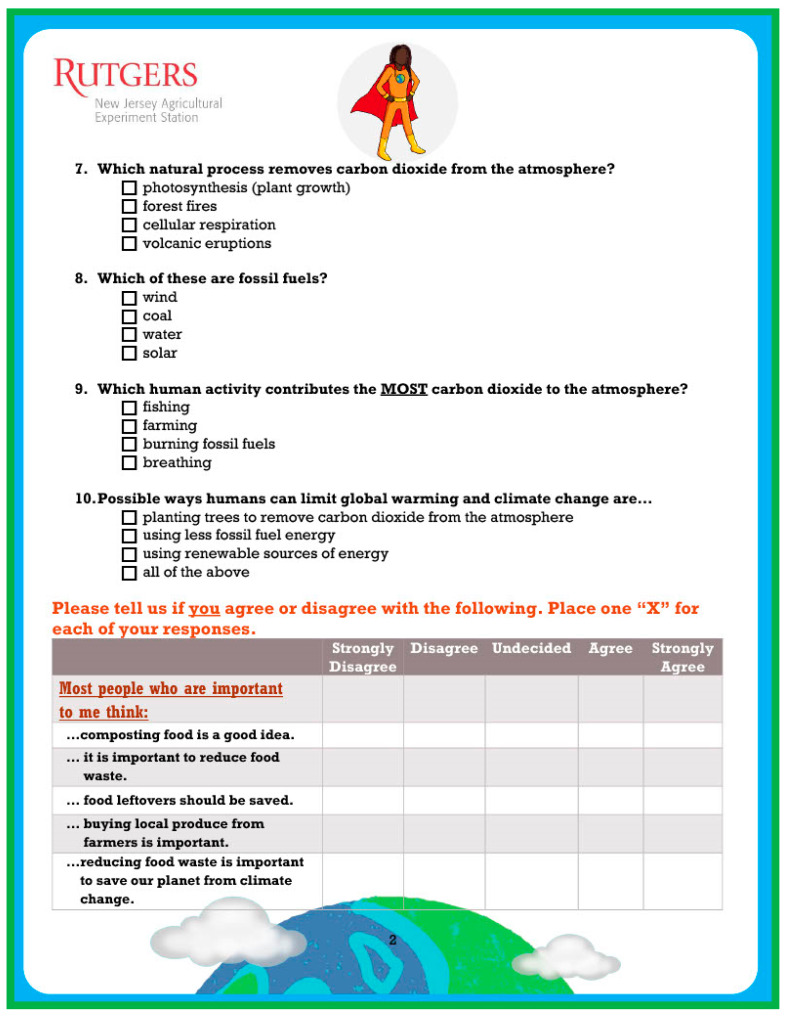

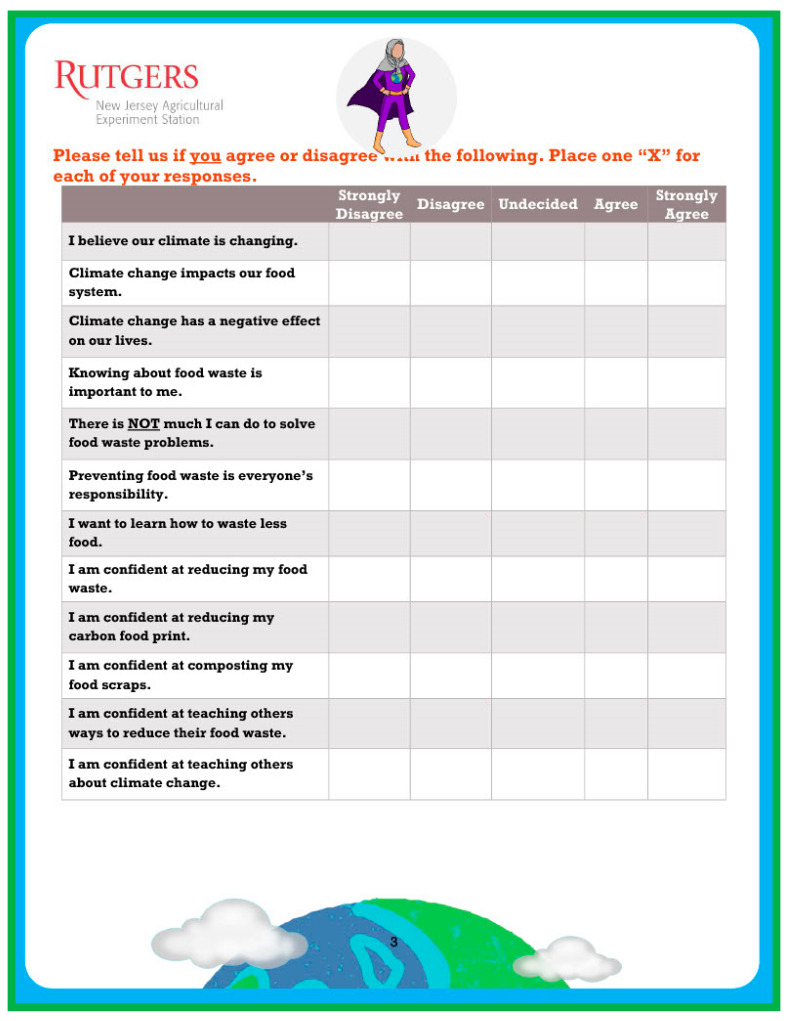

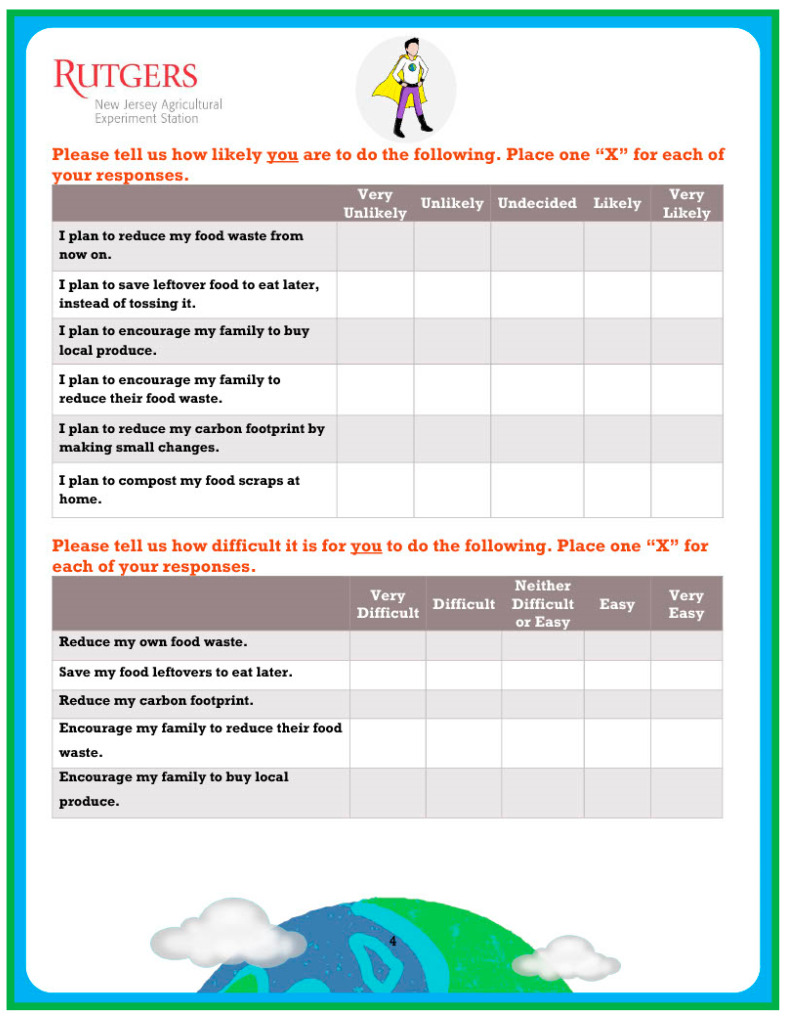

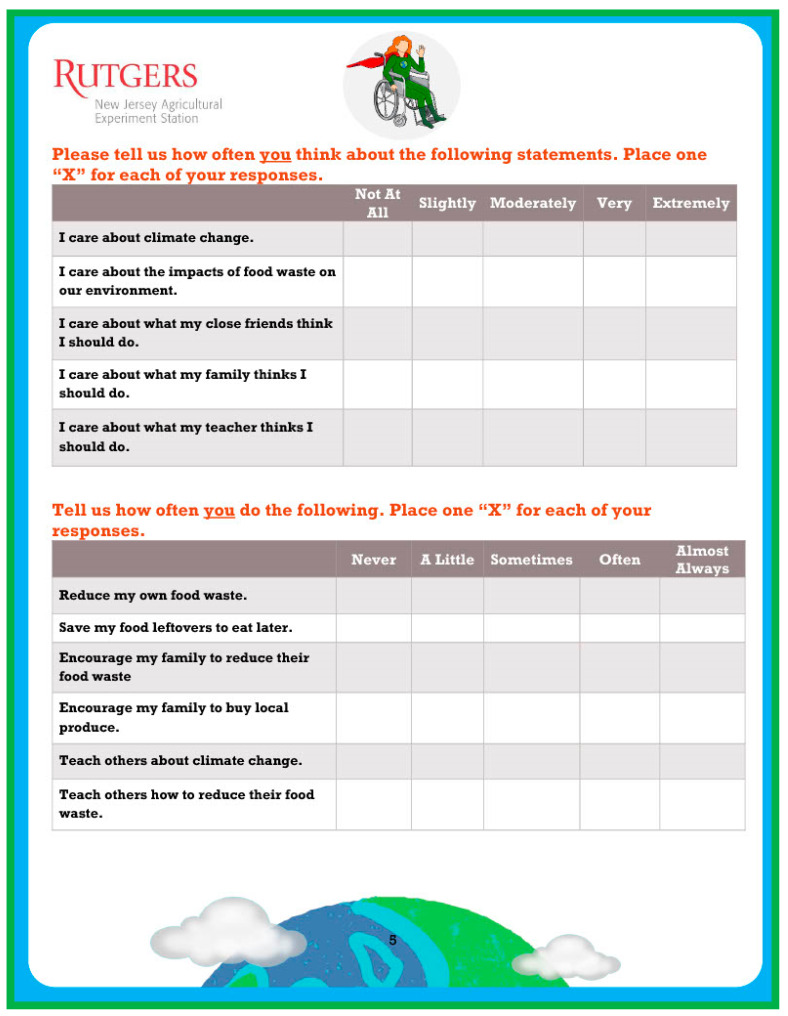

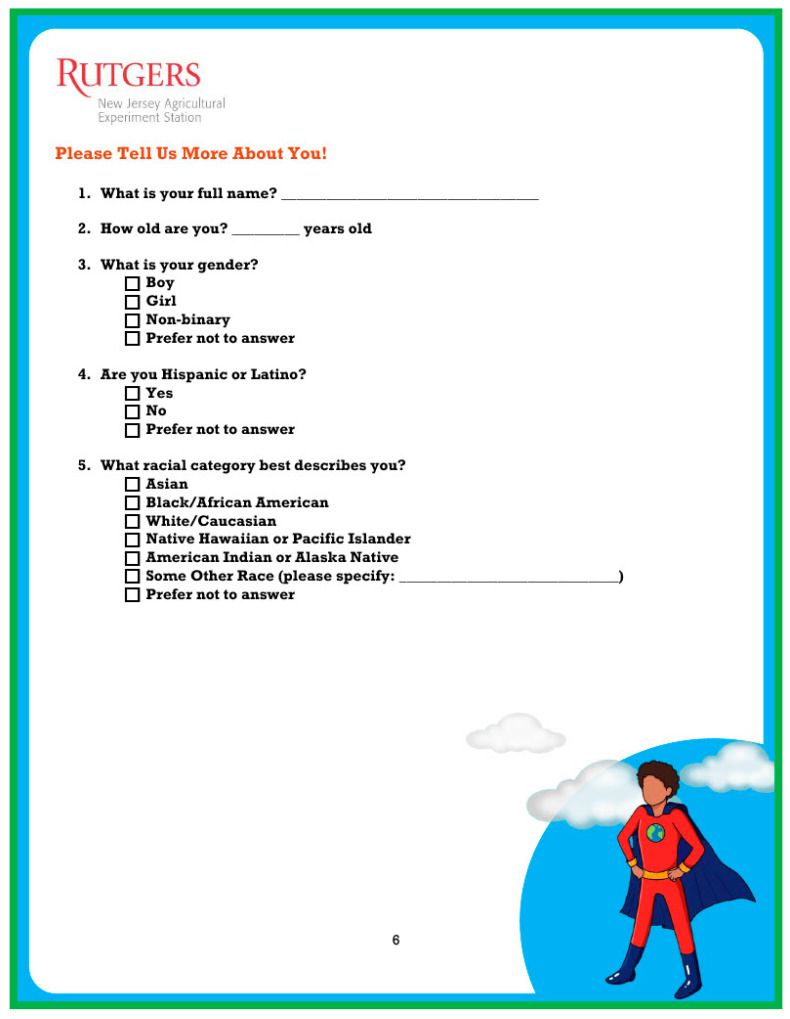
Student Survey.

## Data Availability

The raw data supporting the conclusions of this article will be made available by the authors upon request.

## Appendix A

**Table A1:** NJLNBB Survey Items and Theory of Planned Behavior Scales.

Scale	Response Coding
**Knowledge**	A percentage of total knowledge scores from all questions answered correctly is calculated with higher scores indicating greater knowledge on climate change concepts.
Please select the correct answer for each of the following questions.	
1. Which organisms can help during composting?	1 = honeybees and earthworms; 2 = red ants and beetles; 3 = earthworms and fungi; 4 = earthworms only
2. What is recycled during the process of composting?	1 = glass bottles; 2 = foodwaste; 3 = plastic containers; 4 = aluminum cans
3. Global warming is known as the gradual increase in _______ of the atmosphere.	1 = precipitation; 2 = temperature; 3 = greenhouse gases; 4 = ocean currents
4. The impact on the planet due to global warming is called:	1 = the weather; 2 = the seasons; 3 = climate change; 4 = natural change
5. Earth is currently experiencing…	1 = a warming trend; 2 = a cooling trend; 3 = a period of unchanging climate; 4 = a change in weather
6. Which greenhouse gas is abundant in the Earth’s atmosphere?	1 = nitrogen; 2 = oxygen; 3 = methane; 4 = carbon dioxide
7. Which natural process removes carbon dioxide from the atmosphere?	1 = photosynthesis; 2 = forest fires; 3 = cellular respiration; 4 = volcanic eruptions
8. Which of these options show two types of fossil fuels?	1 = water and natural gas; 2 = oil and coal; 3 = solar and oil; 4 = wind and natural gas
9. Which activity contributes the MOST carbon dioxide to the atmosphere?	1 = fishing; 2 = farming; 3 = burning fossil fuels; 4 = planting trees
10. Possible ways people can limit global warming and climate change are…	1 = planting trees to remove CO_2_ from atmosphere; 2 = leaving TV on during the day; 3 = driving to school instead of walking; 4 = keeping bedroom lights on while at school
**Social Norms (Subjective Norms)**	All items on 5-point Likert scale (1 = Strongly Disagree; 2 = Disagree; 3 = Undecided; 4 = Agree; 5 = Strong Agree). Items are averaged with higher mean scores indicating greater influence received from others to engage in climate-friendly behaviors.
Tell us if you agree or disagree with the following.	
1. Most people who are important to me think composting is a good idea.	
2. Most people who are important to me think it is important to reduce food waste.	
3. Most people who are important to me think buying produce from farmers who live nearby is important	
4. Most people who are important to me think reducing food waste is important to save our planet from climate change.	
**Attitudes**	All items on 5-point Likert scale (1 = Strongly Disagree; 2 = Disagree; 3 = Undecided; 4 = Agree; 5 = Strong Agree). Items are averaged with higher mean scores indicating positive attitudes towards climate-friendly behaviors.
Tell us if you agree or disagree with the following.	
1. I believe our climate is changing.	
2. Climate change effects our food system.	
3. Climate change has a negative effect on our lives.	
4. It is important to me to reduce my own food waste.	
5. Everyone should be responsible in reducing their food waste.	
6. I want to learn how to waste less food.	
**Self-Efficacy**	All items on 5-point Likert scale (1 = Strongly Disagree; 2 = Disagree; 3 = Undecided; 4 = Agree; 5 = Strong Agree). Items are averaged with higher mean scores indicating greater confidence towards adopting climate-friendly behaviors.
Tell us if you agree or disagree with the following.	
1. I am confident at reducing my food waste.	
2. I am confident at composting my food scraps.	
3. I am confident at telling others how they can reduce their food waste.	
4. I am confident at teaching others about climate change.	
**Perceived Behavioral Control**	All items on 5-point Likert scale (1 = Very Difficult; 2 = Difficult; 3 = Neither Difficult or Easy; 4 = Easy; 5 = Very Easy). Items are averaged with higher mean scores indicating greater perceived ease of control over adopting climate-friendly behaviors.
Tell us how difficult it is for you to do the following.	
1. Reduce my own food waste.	
2. Save my food leftover to eat later.	
3. Reduce my GHG emissions.	
4. Encourage my family to reduce their food waste.	
5. Encourage my family to buy produce from farmers that live nearby.	
**Behavioral Intentions**	All items on 5-point Likert scale (1 = Very Unlikely; 2 = Unlikely; 3 = Undecided; 4 = Likely 5 = Very Likely). Items are averaged with higher mean scores indicating greater intentions towards adopting climate-friendly behaviors.
Tell us how likely you plan to do the following.	
1. I plan to reduce my own food waste from now on.	
2. I plan to save leftover food to eat later, instead of tossing it.	
3. I plan to encourage my family to buy produce from farmers that live nearby.	
4. I plan to encourage my family to reduce their food waste.	
5. I plan to reduce my GHG emissions by making small changes.	
6. I plan to compost my food scraps at home.	
**Motivation to Comply**	All items on 5-point Likert scale (1 = Not at all; 2 = Slightly; 3 = Moderately; 4 = Very; 5 = Extremely). Items are averaged with higher mean scores indicating greater motivation towards adopting climate-friendly behaviors.
Tell us how often you think about the following.	
1. I care about climate change.	
2. I care about the impacts of food waste on our environment.	
3. I care about what my close friends think I should do.	
4. I care about what my family thinks I should do.	
5. I care about what my teacher thinks I should do.	
**Climate-Friendly Behaviors**	All items on 5-point Likert scale (1 = Never; 2 = A Little; 3 = Sometimes; 4 = Often; 5 = Almost Always). Items are averaged with higher mean scores indicating greater frequency with engaging in climate friendly behaviors.
Tell us how often you do the following.	
1. Reduce my own food waste.	
2. Save my food leftovers to eat later	
3. Encourage my family to reduce their food waste.	
4. Encourage my family to buy produce from farmers that live nearby.	
5. Teach others about climate change.	
6. Tell others how they can reduce their food waste.	

## References

[1] Canning P., Rehkamp S., Hitaj C., Peters C. (2020). Resource requirements of food demand in the United States. USDA Economic Research Report Number 273.

[2] Gunders D., Bloom J., Berkenkamp J., Hoover D., Spacht A., Mourad M. (2017). Wasted: How America Is Losing up to 40 Percent of Its Food from Farm to Fork to Landfill.

[3] USDA Economic Research Service. https://www.ers.usda.gov/topics/food-nutrition-assistance/food-security-in-the-u-s/key-statistics-graphics/.

[4] National Institute on Minority Health and Health Disparities. https://www.nimhd.nih.gov/resources/understanding-health-disparities/food-accessibility-insecurity-and-health-outcomes.html#chartHouseholds.

[5] ReFed. https://refed.org/.

[6] Buzby J. (2022). Food Waste and Its Links to Greenhouse Gases and Climate Change.

[7] Project Drawdown. https://drawdown.org/solutions.

[8] World Wildlife Fund (2019). Food Waste Warriors: A Deep Dive into Food Waste in US Schools.

[9] US Department of Agriculture. https://www.usda.gov/foodlossandwaste/schools.

[10] Centers for Disease Control and Prevention. https://www.cdc.gov/healthyschools/nutrition/school_nutrition_education.htm.

[11] State of New Jersey. https://www.nj.gov/education/cccs/2020/.

[12] State of New Jersey. https://nj.gov/governor/admin/fl/climate.shtml.

[13] Rousell D., Cutter-Mackenzie-Knowles A. (2020). A systematic review of climate change education: Giving children and young people a ‘voice’ and a ‘hand’ in redressing climate change. Child. Geogr..

[14] Antón-Peset A., Fernandez-Zamudio M., Pina T. (2021). Promoting food waste reduction at primary schools. Sustainability.

[15] Costarelli V., Michou M., Eleni S., Nancy K., Marina S., Katia A., Konstadinos A. (2022). ‘Healthy children, healthy planet’: A pilot school-based educational intervention. Health Educ. J..

[16] Jones M., Dailami N., Weitkamp E., Salmon D., Kimberlee R., Morley A., Orme J. (2012). Food sustainability education as a route to healthier eating: Evaluation of a multi-component school programme in English primary schools. Health Educ. Res..

[17] Kowalewska M.T., Kołłajtis-Dołowy A. (2018). Food, nutrient, and energy waste among school students. Br. Food J..

[18] Nikarvech M., Langen N., Bendisch F., Ziesemer F., Abels S., Schrader U., Fischer D. (2020). The food waste lab: Improved food was reduction behavior through eduction. J. Clean. Prod..

[19] Prescott M.P., Burg X., Metcalfe J.J., Lipka A.E., Herritt C., Cunningham-Sabo L. (2019). Healthy planet, healthy youth: A food systems education and promotion intervention to improve adolescent diet quality and reduce food waste. Nutrients.

[20] Redman E. (2013). Advancing educational pedagogy for sustainability: Developing and implementing programs to transform behaviors. Int. J. Environ. Sci. Educ..

[21] Next Generation Science Standards. https://www.nextgenscience.org/.

[22] Ajzen I. (1991). The theory of planned behavior. Organ. Behav. Hum. Decis. Process..

[23] Langhout R.D., Fernandez J.S., Fetterman D., Kaftarian S.J., Wandersman A. (2014). Empowerment evaluation conducted by fourth- and fifth-grade students. Empowerment Evaluation.

[24] Trott C.D. (2020). Children’s constructive climate change engagement: Empowering awareness, agency, and action. Environ. Educ. Res..

[25] Soorani F., Ahmadvand M. (2019). Determinants of consumers’ food management behavior: Applying and extending the theory of planned behavior. Waste Manag..

[26] Bosnjak M., Ajzen I., Schmidt P. (2020). The Theory of Planned Behavior: Selected recent advances and applications. Eur. J. Psychol..

[27] Ajzen I. (2002). Perceived behavioral control, self-efficacy, locus of control, and the Theory of Planned Behavior. J. Appl. Soc. Psychol..

